# Thermodynamic Model for Hydrogen Production from Rice Straw Supercritical Water Gasification

**DOI:** 10.3390/ma17123038

**Published:** 2024-06-20

**Authors:** Zhigang Liu, Zhiyong Peng, Lei Yi, Le Wang, Jingwei Chen, Bin Chen, Liejin Guo

**Affiliations:** 1 State Key Laboratory of Multiphase Flow in Power Engineering (SKLMF), Xi’an Jiaotong University, No. 28 Xianning West Road, Xi’an 710049, China; 9120030053@jxust.edu.cn (Z.L.); chenbin@xjtu.edu.cn (B.C.); 2 International Institute for Innovation, Jiangxi University of Science and Technology, Ganzhou 341000, China; l-yi@jxust.edu.cn (L.Y.); wang-le@jxust.edu.cn (L.W.); 3 College of Mechanical and Vehicle Engineering, Hunan University, Changsha 410082, China; chenjingwei@hnu.edu.cn

**Keywords:** rice straw, supercritical water gasification, hydrogen, thermodynamic analysis

## Abstract

Supercritical water gasification (SCWG) technology is highly promising for its ability to cleanly and efficiently convert biomass to hydrogen. This paper developed a model for the gasification of rice straw in supercritical water (SCW) to predict the direction and limit of the reaction based on the Gibbs free energy minimization principle. The equilibrium distribution of rice straw gasification products was analyzed under a wide range of parameters including temperatures of 400–1200 °C, pressures of 20–50 MPa, and rice straw concentrations of 5–40 wt%. Coke may not be produced due to the excellent properties of supercritical water under thermodynamic constraints. Higher temperatures, lower pressures, and biomass concentrations facilitated the movement of the chemical equilibrium towards hydrogen production. The hydrogen yield was 47.17 mol/kg at a temperature of 650 °C, a pressure of 25 MPa, and a rice straw concentration of 5 wt%. Meanwhile, there is an absorptive process in the rice straw SCWG process for high-calorific value hydrogen production. Energy self-sufficiency of the SCWG process can be maintained by adding small amounts of oxygen (ER < 0.2). This work would be of great value in guiding rice straw SCWG experiments.

## 1. Introduction

With the leapfrogging of global industry, the massive consumption of fossil energy has resulted in increasing emissions to the environment [1]. The Paris Agreement, a climate change agreement signed by 178 parties from around the world, aims to limit the increase in global average temperature to 2 °C over the pre-industrial period and to work towards limiting the temperature increase to 1.5 °C [2]. In response to this global challenge, China has set a dual-carbon goal of achieving carbon peaking by 2030 and carbon neutrality by 2060. The development and utilization of renewable and clean energy resources, such as biomass, to change the way energy is produced and consumed may be key for building a sustainable energy system to achieve the dual-carbon goal.

Biomass, as a renewable energy source with abundant production and easy access, is mainly composed of cellulose, hemicellulose, and lignin [3,4]. Cellulose is a polysaccharide consisting of glucose monomers linked by β (1,4) glycosidic bonds. Hemicellulose consists of various sugar monomers such as xylose, galactose, and glucose, which are easily hydrolyzed. Lignin is a polymer with a three-dimensional network structure formed by three kinds of phenylpropane units connected to each other through ether and carbon–carbon bonds. Rice straw, as a typical representative of agricultural biomass residues, has a global annual production of 800 million tons, of which China is the largest rice producer [5]. The efficient use and conversion of such abundant and accessible biomass resources has been a hot research topic. Resource utilization technologies for biomass include two broad categories: biochemical conversion and thermochemical methods [6,7,8,9]. Biochemical conversion, biogas technology, and hydrolytic fermentation use the metabolism of micro-organisms, such as bacteria, to produce combustible gases or liquid fuels to produce ethanol, hydrogen, etc. However, biochemical conversion has the shortcomings of low conversion efficiency, low end-product yield, difficulty in low-cost scale-up of the conversion process, and the need for secondary treatment of residual substrates. Conventional thermochemical conversion methods also suffer from treatment instability and low conversion efficiency because of the differences in the composition of different biomasses and their high water content. Therefore, there is an urgent need to develop new biomass conversion and utilization technologies.

Recently, the emerging supercritical water gasification (SCWG) technology has taken advantage of the unique physicochemical properties of supercritical water to explore a new highly efficient and clean biomass thermochemical conversion route [10,11,12]. Supercritical water (SCW) is water at temperatures and pressures exceeding 374.2 °C and 22.1 MPa, respectively. The physicochemical properties of supercritical water are significantly different from those of normal water. Supercritical water has a low viscosity and dielectric constant, and its solubility tends to be similar to that of organic solvents while its diffusion coefficient tends to be similar to that of gases [13,14]. The ability of supercritical water to be miscible with organics and gases in any ratio allows reactions to be conducted under homogeneous conditions, greatly facilitating reaction rates [15,16]. Supercritical water gasification biomass technology converts biomass into a highly concentrated hydrogen-rich gas mixture at lower temperatures and reducing conditions, and also avoids the generation of pollutants such as nitrogen compounds, sulfides, and suspended particles during combustion and conventional gasification processes [17,18]. The technology has attracted increasing attention at home and abroad due to many advantages such as high energy conversion efficiency and a wide range of feedstock adaptations.

Yanik et al. [19] conducted experiments using a batch high-pressure reactor at 500 °C with eight different biomasses, such as corn stover and cotton stalks, as feedstock. The amino acid, protein, and oil content of different crops affected the hydrogen yield, which ranged from 4.05 to 4.65 mol/kg of biomass. Williams et al. [20] investigated the SCWG characteristics of cellulose, starch, glucose, and cassava waste with a batch high-pressure reactor. Glucose had the highest hydrogen yield and cassava waste had the lowest. Although both starch and cellulose are polymers of glucose, cellulose produces more hydrocarbons during the gasification process, while starch produces more H_2_, CO, and oils. Lu et al. [21] used nickel catalysts to achieve efficient gasification of glucose in supercritical water. The addition of Ni/γAl_2_O_3_ and Ni/CeO_2_-γAl_2_O_3_ catalysts can significantly improve the hydrogen yield and selectivity, but carbon accumulation and coking may lead to catalyst deactivation. It was noted that Ce in Ni/CeO_2_-γAl_2_O_3_ catalysts had an inhibitory effect on carbon accumulation and coking. Experimental research can provide reliable data to advance technology, but it can also be expensive in terms of time and resources, especially to capture the effects of multiple parameters. The development of appropriate theoretical models may reveal SCWG reaction principles without being constrained by time and physical limitations.

So far, two approaches have been employed for theoretical modeling, namely the stoichiometric (kinetic) approach and the non-stoichiometric (thermodynamic) approach [22,23]. The stoichiometric approach relies on detailed and accurate reaction networks to quantify the reaction process by obtaining the defined reaction parameters. However, the complexity of biomass composition makes it difficult to determine its reaction network in the SCWG process. The non-stoichiometric approach based on the Gibbs free energy minimization principle is more advantageous in predicting the product distribution at reaction equilibrium than the stoichiometric approach. Gibbs free energy, a thermodynamic function introduced as a way of determining the direction in which a process proceeds, is an important concept and method in chemical thermodynamics. It refers to the portion of a system’s reduced internal energy that can be converted to external work in a given thermodynamic process. Tang et al. [24] analyzed the chemical equilibrium of methanol, glucose, cellulose, and real biomass in supercritical water based on the Gibbs free energy minimization principle and the choice of the Peng–Robinson (PM) equation of state. The developed model is a powerful tool for analyzing the SCWG process due to its ability to effectively predict the actual gasification process. Castello et al. [25] calculated the product distribution of biomass in supercritical water at the Gibbs free energy minimum using the PM equation. Total gasification of biomass in supercritical water is possible because coke may not be produced under actual biomass concentrations (<40 wt%) due to thermodynamic constraints.

Although progress has been made in current research on the thermodynamics of supercritical water gasification, more attention needs to be paid to the calculation of the thermodynamics of supercritical water gasification of biomass, especially complex real biomass. It is also attractive to focus on the reaction heat load of biomass supercritical water because the production of hydrogen from multiphase reaction processes at high temperatures and pressures may require heat absorption. The present work developed an SCWG thermodynamic model based on the Gibbs free energy minimization principle using rice straw as a typical representative of real biomass. The product distribution characteristics of rice straw SCWG in the equilibrium process were investigated for different operating parameters (temperature, pressure, and biomass concentration). The heat duty of the rice straw SCWG process and the variation of the calorific value of the products were investigated under different oxygen equivalence ratios.

## 2. Modeling Approach

### 2.1. Thermodynamic Equilibrium Method

It is extremely difficult to consider all liquid-phase products in a thermodynamic model because the liquid-phase intermediates of a real biomass supercritical water gasification process are numerous and complex [26,27]. Note that the liquid-phase organic products of gasification generally represent a relatively small mass fraction of the total gasification products, making them less critical and less necessary [28,29,30]. Therefore, the liquid-phase products after gasification were not considered in order to simplify the difficulty of model calculation by choosing H_2_O, H_2_, CO, CH_4_, CO_2_, and C as the final possible gasification products in this paper. A thermodynamic analytical model for the equilibrium composition of rice straw SCWG products was developed. The relevant physical properties of rice straw are shown in Table 1.

When the chemical reaction reaches equilibrium, the Gibbs free energy of the whole reaction system reaches the minimum. Thus, the following thermodynamic model was established in this paper using the principle of minimum Gibbs free energy (*G*):

Objective function:

(1) minG

Subject to:

(2) $$\overset{N}{\underset{i}{\sum }}\beta_{e,t}n_{t}=\beta_{e}\;\;\left(e=C,H,O\right)$$

(3) $$n_{t}\geq 0\;\;\left(t=H_{2}O,{\;H}_{2},\;\mathrm{CO},\;{\mathrm{CH}}_{4},\;{\mathrm{CO}}_{2},\;C\right)$$

where $\beta_{e,t}$ is the number of atoms of the *e* element in the *t* compound; $n_{t}$ is the number of moles of the *t* compound when the reaction reaches equilibrium; $\beta_{e}$ is the number of moles of the *e* element in the reactant.

Ortiz et al. [31] used four equations of state, including the Predictive Soave–Redlich–Kwong Equation (PSRK), the Soave–Redlich–Kwong Equation (SRK), the Peng–Robinson Equation (PR), and the Peng–Robinson–Boston–Mathias Equation (PR-BM), for the calculation of supercritical fluid properties. The PR and the PR-BM equations can produce a very good fit. Qi et al. [32] employed the PR and PR-BM equations to perform the calculation of the products of supercritical water gasification equilibrium of black liquor (lignin). The results showed that the product equilibrium calculated by the PR-BM equation was supported by the experimental results. The PR-BM equation, suitable for both nonpolar and weakly polar systems in the supercritical and subcritical regions, is chosen to calculate the physical properties in this paper. The equation has also been widely used by many scholars in the calculation process of relevant thermodynamic parameters [33,34,35].

(4) $$P=\frac{RT\;}{v-b}-\frac{a}{v\left(v+b\right)+b\left(v-b\right)}$$

where *v* is the mole volume in m^3^/mol. *a* and *b* are the temperature-independent attraction and repulsion parameters, which are calculated by Equations (5) and (6).

(5) $$a=\frac{0.45724R^{2}T_{c}^{2}\;}{P_{c}}\alpha \left(T\right)$$

(6) $$b=\frac{0.07780RT_{c}\;}{P_{c}}$$

where $T_{c}$ and $P_{c}$ are the critical temperature in K and critical pressure in Pa, respectively. α(T) is a temperature-dependent *α*-function. The equation for the Boston–Mathias *α*-function, a piecewise function, can be expressed as Equation (7) at supercritical conditions [36].

(7) $$\alpha \left(T\right)=\exp\left[c_{e}\left(1-{\left(T_{r}\right)}^{d}\right)\right],\;\;\;T_{r}=\frac{T\;}{T_{c}}>1$$

(8) $$c_{e}=\frac{m\;}{d}$$

(9) $$d=1+\frac{m\;}{2}$$

(10) $$m=0.37464+1.54226\omega -0.26992\omega^{2}$$

where *ω* is an acentric factor.

The supercritical water gasification process of rice straw was simulated in Aspen plus. An RStoic module and an RGibbs module were used to calculate the product composition. The RStoic module uses a FORTRAN subroutine to decompose rice straw based on its elemental composition. Substances decomposed in the RStoic module finish the SCWG process in the RGibbs module, which is based on the Gibbs free energy minimization principle to predict the product equilibrium composition.

### 2.2. Model Validation

**Figure 1:**
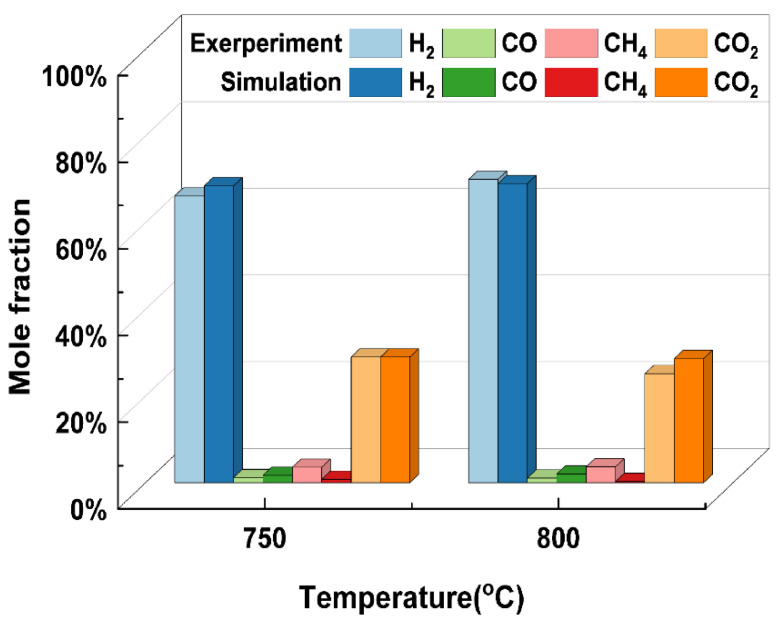
Comparison of simulation results with the experimental results under different gasification temperatures (glycerol, 24.1 MPa, 5 wt%).

It is first necessary to verify the accuracy of the model before using the thermodynamic model developed above for further analyses of the reliability of the results obtained from the thermodynamic model. Many researchers have carried out supercritical water gasification experiments using glycerol as a model compound for biomass [31,37]. This paper selected glycerol SCWG conducted by Byrd et al. [38] as the material for validation due to the lack of access to experimental work on the SCWG of rice straw. Figure 1 shows that the simulation results can be well-supported by the experimental results to indicate the feasibility of the model. It is worth noting that liquid intermediates are ignored in the SCWG thermodynamic model developed in this paper. Glycerol with a simple structure is easily and totally gasified in supercritical water with few liquid phase products. For real biomass such as rice straw, the SCWG process might generate intermediate products. Therefore, the model may have some limitations, and more experimental data on rice straw need to be obtained for future improvements.

## 3. Results and Discussion

### 3.1. Effects of Operating Conditions on SCWG of Rice Straw

The supercritical water gasification process of water rice straw may undergo a series of reactions such as hydrolysis, pyrolysis, liquefaction, and gasification. The steam-reforming reaction, water–gas shift reaction, and methanation reaction during the SCWG of organics are accepted by researchers [39,40,41] as the main reactions, as shown in Equations (11)–(13). Gas molar fraction (14) and gas yield (15) were chosen as indicators to evaluate the gasification results in this paper.

(11) $$C+H_{2}O\to \mathrm{CO}+H_{2}\;\Delta H_{298\;k}=131.29\;\mathrm{kJ}/\mathrm{mol}$$

(12) $$\mathrm{CO}+H_{2}O↔{\mathrm{CO}}_{2}+H_{2}\;\Delta H_{298\;k}=-41.17\;\mathrm{kJ}/\mathrm{mol}$$

(13) $$\mathrm{CO}+3H_{2}↔{\mathrm{CH}}_{4}+H_{2}O\;\Delta H_{298\;k}=-206.10\;\mathrm{kJ}/\mathrm{mol}$$

(14) $$\mathrm{Gas}\;\mathrm{molar}\;\mathrm{fraction}=\frac{\mathrm{the}\;\mathrm{mole}\;\mathrm{of}\;\mathrm{gaseous}\;\mathrm{product}}{\mathrm{the}\;\mathrm{molar}\;\mathrm{number}\;\mathrm{of}\;\mathrm{all}\;\mathrm{the}\;\mathrm{gaseous}}\times 100\%$$

(15) $$\mathrm{Gas}\;\mathrm{yield}=\frac{\mathrm{the}\;\mathrm{mole}\;\mathrm{of}\;\mathrm{gaseous}\;\mathrm{product}}{\mathrm{the}\;\mathrm{mass}\;\mathrm{of}\;\mathrm{the}\;\mathrm{rice}\;\mathrm{straw}}\left(\mathrm{mol}/\mathrm{kg}\right)$$

#### 3.1.1. Effect of Temperature

**Figure 2:**
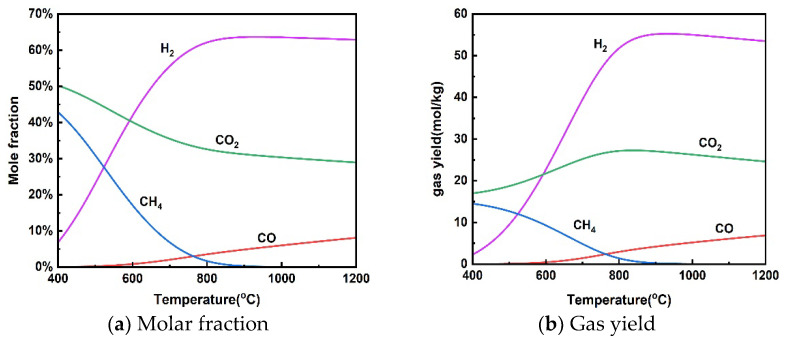
Effect of temperature on product distribution (25 MPa, 10 wt% rice straw).

Figure 2 shows the effect of reaction temperatures in the range of 400–1200 °C on gas molar fraction and gas yield at a pressure of 25 MPa and a rice straw concentration of 10 wt%. The effect of temperature on H_2_ production from the supercritical water gasification of rice straw is roughly divided into two stages, including a rapid increase in H_2_ at 400–800 °C and a smooth change from 800–1200 °C. Kang et al. [42] found that the average hydrogen production of various biomass SCWGs increased from 0.32 mmol/g to 1.85 mmol/g as the temperature increased from 450–650, but the trend of the average hydrogen production with increasing temperature slowed down when the temperature was higher than 550 °C. There was a significant decrease in CH_4_ yield in the gasification process and an increase in H_2_ yield when the temperature was increased from 400 to 800 °C. This change in gas composition can be explained by the properties of the reactions performed in supercritical water gasification. The methanation reaction (13) was inhibited at higher temperatures because it is exothermic. On the other hand, the reforming of methane was enhanced because it is a heat-absorbing reaction. Both processes result in a decrease in CH_4_ production and an increase in H_2_ production. Meanwhile, the enhancement of steam reforming (11) of the heat-absorption reaction with increasing temperature leads to an increase in H_2_ production. In addition, the water–gas shift reaction (12) was also inhibited at high temperatures due to its exothermic properties, reducing the conversion of CO to CO_2_ and H_2_. Therefore, CO content increased with increasing temperature. Lu et al. [43] found that temperature has the greatest effect on biomass supercritical water gasification using the orthogonal method because high temperatures support free radical reactions.

It can be seen from Figure 2 that the gas yield does not change much. In particular, H_2_ yield showed a slight decrease when the temperature was above 800 °C. It is also taken into account that overly high temperatures increase the cost of the gasification process and lead to material limitations. The supercritical water gasification temperature of rice straw should not exceed 800 °C.

#### 3.1.2. Effect of Pressure

**Figure 3:**
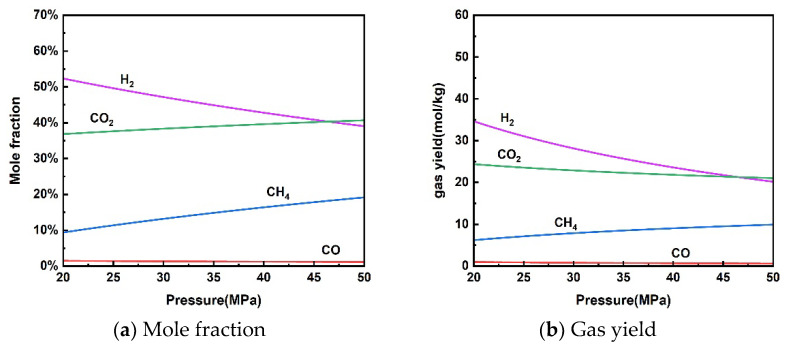
Effect of pressure on product distribution (650 °C, 10 wt% rice straw).

The effect of reaction pressure on the thermodynamic equilibrium of rice straw gasification in supercritical water is presented in Figure 3. As the pressure increased from 20 MPa to 50 MPa, the molar fraction of H_2_ and gas yield decreased from 52.33% and 34.62 mol/kg to 39.02% and 20.18 mol/kg, respectively. The molar fraction of CH_4_ and gas yield increased from 9.37% and 6.20 mol/kg to 19.16% and 9.91 mol/kg, respectively. Gas yields of CO and CO_2_ showed a slight decline. An increase in pressure promotes a shift in gas volume in the direction of decrease according to Le Chatelier’s principle of equilibrium shift. Therefore, the methanation reaction (13) moved in a positive direction making H_2_ production decrease and CH_4_ production increase. CO and CO_2_ changed very little due to the minimal impact on the water–gas shift reaction.

The effect of pressure on the supercritical water gasification process is more complex due to two competing reactions [30]. Although increased pressure promotes the hydrolysis of organic matter in supercritical water, it also inhibits free radical reactions to produce abundant gases. Experiments by Bai et al. [44] and Fan et al. [45] showed no significant effect of pressure on the supercritical water gasification process. Note that high pressures can increase material costs and operational risks. Therefore, it is only necessary to ensure that the pressure is slightly higher than the critical point to utilize the performance of supercritical water during the actual SCWG process of rice straw.

#### 3.1.3. Effect of Rice Straw Concentration

**Figure 4:**
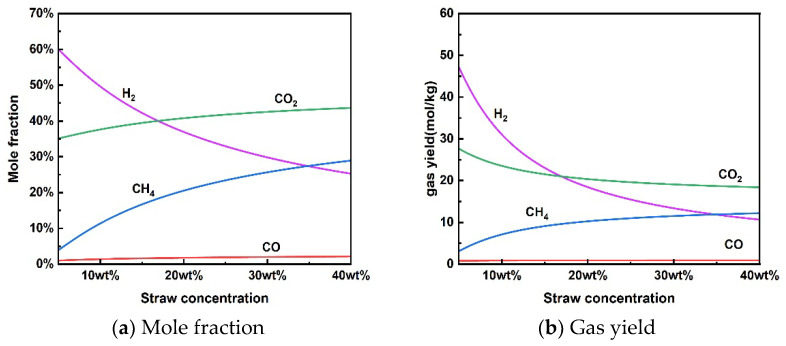
Effect of rice straw concentration on product distribution (650 °C, 25 MPa).

Figure 4 shows the reaction characteristics for the supercritical water gasification of rice straw at different concentrations. It is worth noting that no coke was produced in the concentration range examined under thermodynamic constraints. The results showed that the increase in concentration also had an inhibitory effect on H_2_ production by supercritical water gasification, which decreased the mole fraction and gas yield of H_2_. The opposite trend was observed for the molar fraction and gas yield of CH_4_. The increase in concentration increased the CH_4_ content in the produced gas. For a given temperature of 650 °C and pressure of 25 MPa, H_2_ was the most abundant gas at lower rice straw concentrations (<17 wt%). The gas yield of CH_4_ was higher than that of H_2_ when the concentration of rice straw exceeded 35 wt%. Thus, the increase in high rice straw concentration may facilitate the methanation reaction for the conversion of H_2_ to CH_4_ under thermodynamic constraints [46]. Huelsman et al. [47] investigated the gasification mechanism of different concentrations of phenol in supercritical water. High concentrations of phenol reduced hydrogen yield due to polymerization reactions, leading to deposition and coking. From a kinetic point of view, less water in the reactants may limit the contact between the material particles and water to reduce the reaction rate of SCWG, which is not conducive to total gasification [48].

Although rice straw SCWG can increase the treatment capacity at high rice concentrations, the steam-reforming reaction for hydrogen production is inhibited. At the same time, high-concentration rice straw slurry pulping is also a challenge due to the need for better fluidity and homogeneity in the input system. From the point of view of hydrogen production, the concentration of rice straw for supercritical water gasification of rice straw may not exceed 17 wt% at a temperature of 650 °C and a pressure of 25 MPa.

### 3.2. Analysis of Reaction Heat Duty and Higher Heating Value (HHV) of Gaseous Products

Clarifying the energy changes in the rice straw SCWG gasification process can facilitate energy matching to accelerate the reaction process, of which the reaction heat duty is an important indicator. Consumption of some of the hydrogen-rich gas can provide energy to the gasification process, as shown in Equations (16)–(18). This section analyzed the heat duty of the gasification process as well as the HHV of the gaseous products under different variations of reaction parameters by adding different levels of oxygen, i.e., the oxygen equivalence ratio (ER). The ER can be calculated using Equation (19), if the chemical formula of rice straw is assumed to be C_x_H_y_O_z_, according to elemental analysis of mass fractions. The simulation was calculated assuming an isothermal process and focusing only on the reaction process without including the heating process of the material and the preheated water.

(16) $$H_{2}+\frac{1}{2}O_{2}\to H_{2}O\;\Delta H_{298\;k}=-285.8\;\mathrm{kJ}/\mathrm{mol}$$

(17) $$\mathrm{CO}+\frac{1}{2}O_{2}\to {\mathrm{CO}}_{2}\;\Delta H_{298\;k}=-283.1\;\mathrm{kJ}/\mathrm{mol}$$

(18) $${\mathrm{CH}}_{4}+2O_{2}\to {\mathrm{CO}}_{2}+2H_{2}O\;\Delta H_{298\;k}=-889.6\;\mathrm{kJ}/\mathrm{mol}$$

(19) $$ER=\frac{n_{O_{2}}}{(\frac{x}{12}+\frac{y}{4}-\frac{z}{32}{)\;\times \;m}_{feedstock}}$$

(20) $$\begin{array}{c} \Delta H(P,\;T)=\Delta H_{298\;k}^{0}+\overset{\;}{\underset{s}{\sum }}n_{s}\left[H_{s}\left(P,\;T\right)-H_{s}^{0}\right]-\overset{\;}{\underset{k}{\sum }}n_{k}\left[H_{k}\left(P,\;T\right)-H_{k}^{0}\right] \end{array}$$

(21) $$\begin{array}{c} \Delta H_{298\;k}^{0}=\overset{\;}{\underset{s}{\sum }}n_{s}{HHV}_{s}-\overset{\;}{\underset{k}{\sum }}n_{k}{HHV}_{k} \end{array}$$

**Figure 5:**
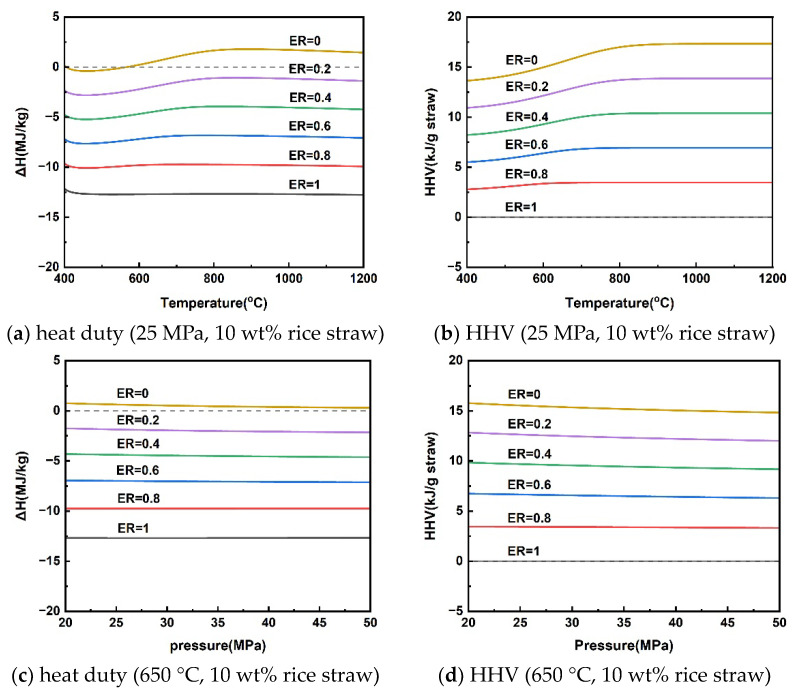

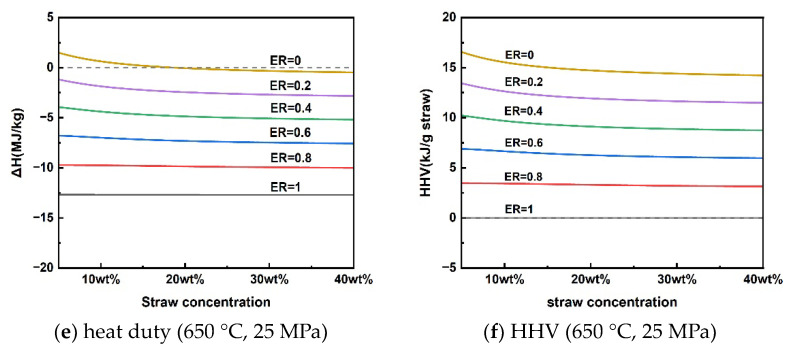
Reaction heat duty and HHV of gaseous products under operating parameters.

The reaction heat duty of rice straw SCWG and the HHV of the gas products at different temperatures for a pressure of 25 MPa and a rice straw concentration of 10 wt% are given in Figure 5a,b. Rice straw supercritical water gasification reaction heat duty was subject to shifts in heat absorption and exothermic properties at different gasification temperatures. Rice straw SCWG was bounded by a reaction temperature of 565 °C; below 565 °C was an exothermic process, and above 565 °C was an absorptive process. This is because the increased gaseous products of high calorific value at high temperatures make it necessary for the rice straw SCWG to absorb more heat. As the pressure increased from 20 MPa to 50 MPa, the rice straw SCWG process was heat-absorbing, showing a decrease in the heat of reaction from 0.75 kJ/g to 0.30 kJ/g. The HHV of the gaseous products also decreased slowly due to the decrease in the production of hydrogen with a high calorific value. Figure 5e,f shows the trends of reaction heat duty and the HHV of gaseous products for rice straw concentrations in the range of 5–40 wt%. The low concentration can favor the production of H_2_ to increase the HHV of the gas products, making rice straw SCWG a heat-absorbing process, while the SCWG process was exothermic due to the inhibition of hydrogen production when the concentration of rice straw exceeds 18.56 wt%. A similar trend was observed in the results of reaction heat load calculations for the supercritical water gasification of diesel fuel by Xu et al. [49]. This depends mainly on the difference between the calorific value of the gas product and the calorific value of the feedstock in different conditions.

From the above analysis, there is a conversion of heat absorption and exothermic processes in the supercritical water gasification of rice straw. The effect of these operating parameters on the absorptive and exothermic conversion depends mainly on the HHV of the gaseous products, where hydrogen is the most critical because of its high calorific value. In other words, the supercritical water gasification process of rice straw for the purpose of hydrogen production is heat-absorbing. It can be seen from Figure 5 that the conversion of the process from heat absorption to exothermic can be achieved by slightly inputting a little oxygen elimination into the supercritical water gasification process. It means that releasing part of the calorific value of the hydrogen-rich gas can achieve energy self-sufficiency for the SCWG process. The energy self-sufficiency of this approach may effectively reduce the consumption of high-quality electrical energy to sustain the SCWG gasification process [50,51].

## 4. Conclusions

This paper presented a thermodynamic analysis of the SCWG process for rice straw treatment. The chemical equilibrium calculations for the whole process were based on the Gibbs free energy minimization principle. The PR-BM equation of state was used to describe the physical properties of a substance in a supercritical state. The gas mole fraction, gas yield, reaction heat duty, and HHV of gas products were investigated for rice straw SCWG at temperatures of 400–1200 °C, pressures of 20–50 MPa, and rice straw concentrations of 5–40 wt%. Note that the absence of coke production under the conditions examined proves the feasibility of total gasification. Higher reaction temperatures can increase hydrogen yield and decrease methane yield due to the promotion of the steam-reforming reaction of methane. High reaction pressures may shift the equilibrium towards lower gas production, inhibiting free radical reactions to reduce hydrogen production. The increased concentration of rice straw was not favorable to SCWG for hydrogen production because the reduced water concentration limited the steam-reforming reaction. Rice straw SCWG targeting hydrogen production is overall a heat-absorbing process. Achieving energy self-sufficiency of the rice straw SCWG process can be performed with small amounts of oxygen (ER < 0.2) input by partial consumption of gas products to provide the heat duty required for gasification. Current thermodynamic models usually do not consider liquid-phase intermediates due to their complexity, but the efficient decomposition of intermediates is a bottleneck for total gasification. Future research may identify key kinetic models for the development of intermediates by experimental studies, combining the two models to improve their applicability and predictive robustness.

## Figures and Tables

**Table 1:** Properties of rice straw (^a^ by difference).

Elemental Analysis ^a^ (wt%, Dry Ash-Free Basis)					Proximate Analysis (wt%, Air-Dry Basis)				LHV (MJ/kg)
C	H	N	S	O ^a^	Moisture	Ash	Volatile Matter	Fixed Carbon	
41.17	5.94	0.97	0.12	51.8	8.09	9.10	68.22	14.59	13.79

## Data Availability

Data are contained within the article.
